# Supplementary figures and images for: The impact of SARS-CoV-2 infection on renal function in patients with biopsy-proven kidney diseases
Source: PLoS One. 2023 Dec 22;18(12):e0296168. doi: 10.1371/journal.pone.0296168 (PMC10745175; doi:10.1371/journal.pone.0296168)

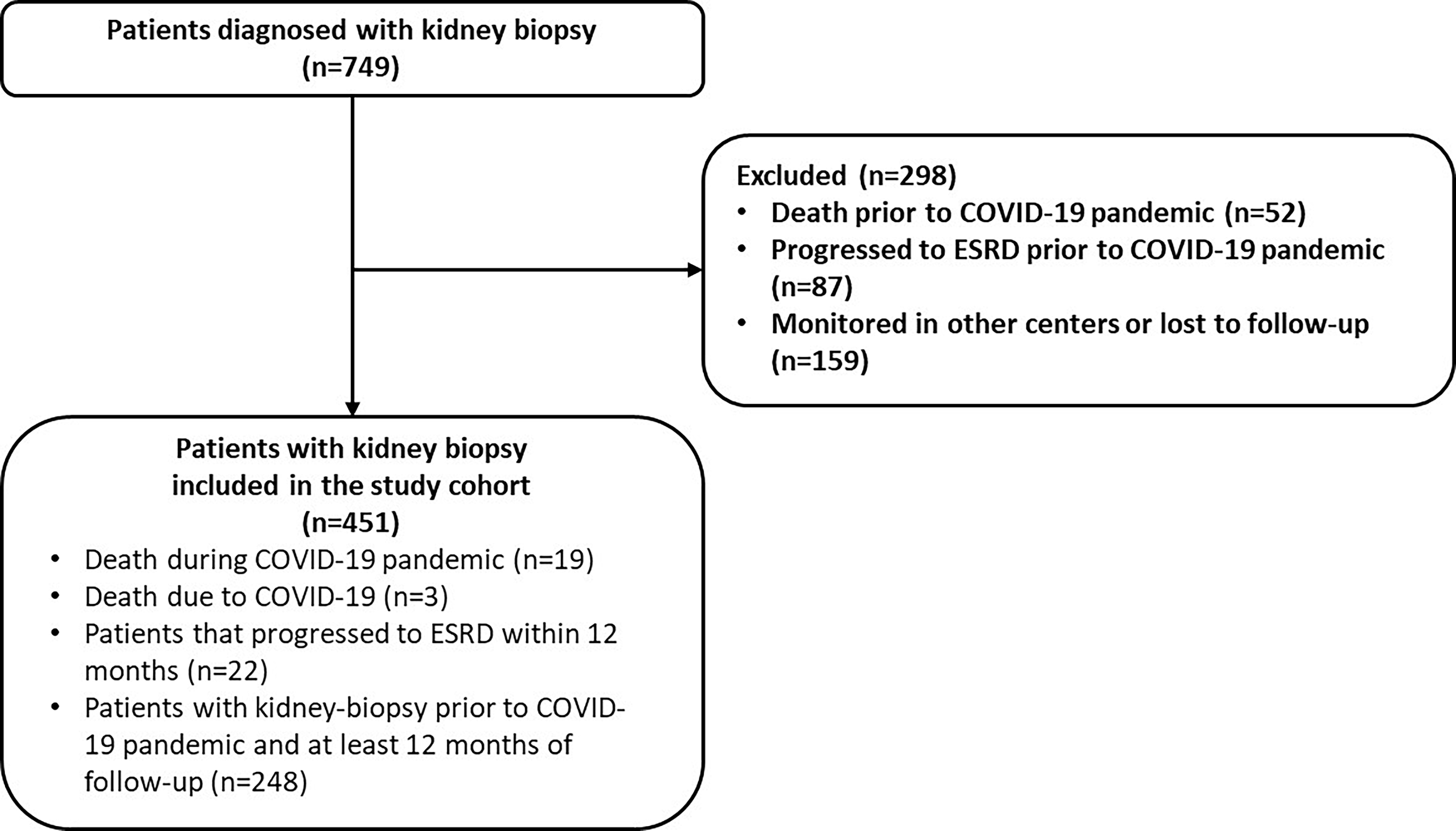

Supplement: S1 Fig — (TIF) [file pone.0296168.s001.tif]

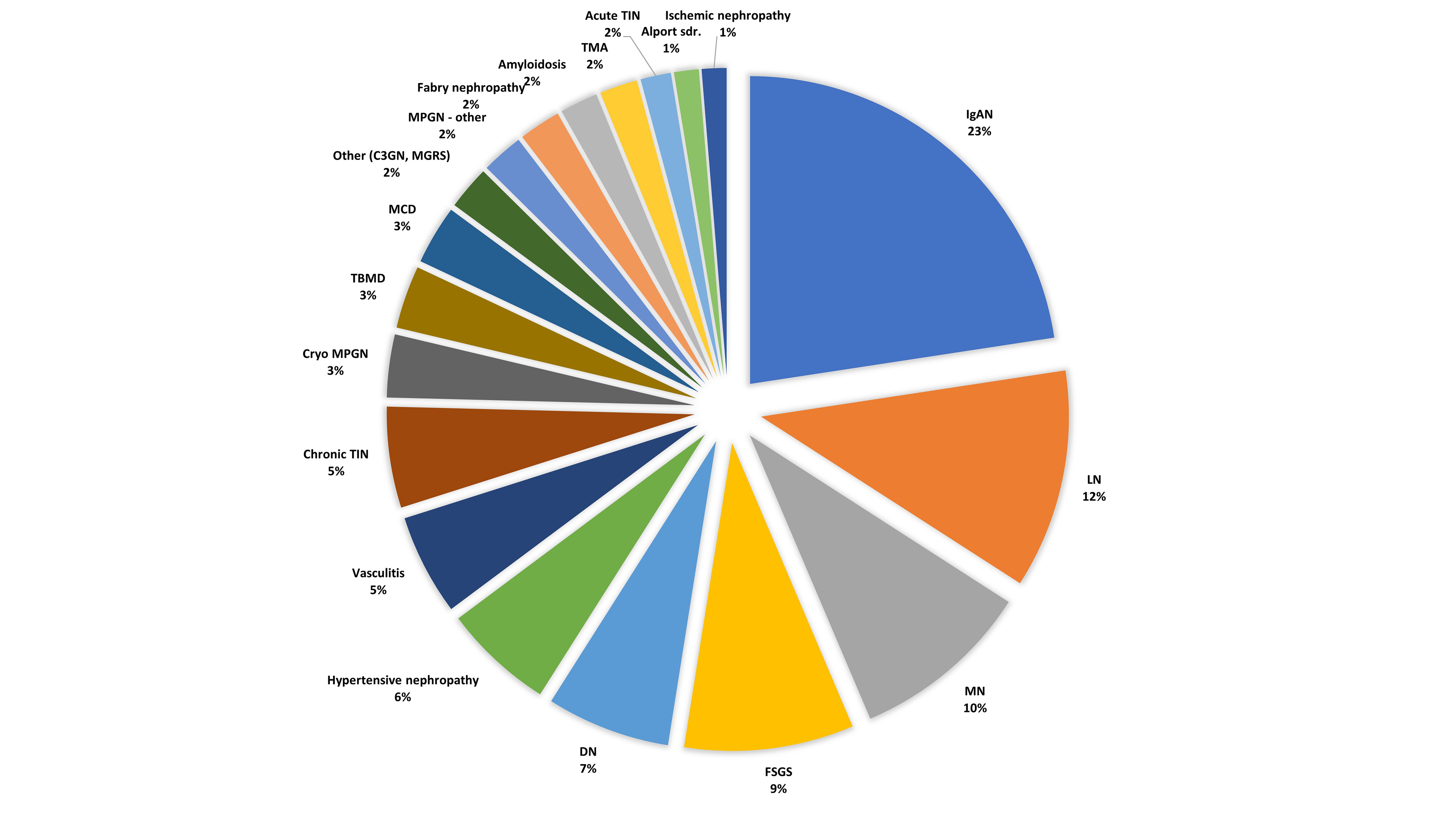

Supplement: S2 Fig — (TIF) [file pone.0296168.s002.tif]
