# Supplementary material for: The impact of SARS-CoV-2 infection on renal function in patients with biopsy-proven kidney diseases
Source: PLoS One. 2023 Dec 22;18(12):e0296168. doi: 10.1371/journal.pone.0296168 (PMC10745175; doi:10.1371/journal.pone.0296168)
Supplement: S1 Table — (DOCX) [file pone.0296168.s003.docx]

**S1 Table. Etiology of renal disease.**

| **Variable** | **Value** |
| --- | --- |
| IgA nephropathy | 102 (22.6%) |
| Membranous nephropathy | 43 (9.5%) |
| Focal and segmental glomerulosclerosis | 40 (8.9%) |
| Minimal change disease | 14 (3.1%) |
| Cryoglobulinemic MPGN | 15 (3.3%) |
| MPGN – other etiologies | 10 (2.2%) |
| Lupus nephritis | 52 (11.5%) |
| Amyloidosis and other fibrillary GN | 9 (2%) |
| Thin basement membrane disease | 15 (3.3%) |
| Alport syndrome. | 6 (1.3%) |
| Fabry nephropathy | 12 (2.2%) |
| Diabetic nephropathy | 29 (6.5%) |
| Ischemic nephropathy | 6 (1.3%) |
| Hypertensive nephropathy | 26 (5.8%) |
| Thrombotic microangiopathy | 9 (2%) |
| Systemic vasculitis | 24 (5.3%) |
| Acute TIN | 7 (1.6%) |
| Chronic TIN | 24 (5.3%) |
| Other nephropathies (C3GN, MGRS, etc.) | 10 (2.3%) |

***Abbreviations:*** *MPGN, membrano-proliferative glomerulonephritis; GN, glomerulonephritis; TIN, tubulointerstitial nephritis; C3GN, C3 glomerulonephritis; MGRS, monoclonal gammopathy of renal significance.*
