# Supplementary material for: The impact of SARS-CoV-2 infection on renal function in patients with biopsy-proven kidney diseases
Source: PLoS One. 2023 Dec 22;18(12):e0296168. doi: 10.1371/journal.pone.0296168 (PMC10745175; doi:10.1371/journal.pone.0296168)
Supplement: S2 Table — (DOCX) [file pone.0296168.s004.docx]

**S2 Table. Sub-analysis according to the severity of COVID-19.**

| **Variable** | **No COVID-19** | **Mild-Moderate**  **COVID-19** | **Severe COVID-19** | **p-value** |
| --- | --- | --- | --- | --- |
| Number of patients | 331 | 70 | 31 |  |
| eGFR at study onset (mg/dl) | 53.1 ± 29.2 | 54 ± 32.8 | 46.1 ± 27.4 | 0.4 |
| eGFR at 12 months (mg/dl) | 56.4 ± 31 | 53 ± 32.8 | 41 ± 30.3 | 0.02 |
| eGFR at last FU (mg/dl) | 56.7 ± 32.6 | 56.8 ± 38.8 | 40.8 ± 32.7 | 0.03 |
| Total eGFR change (ml/min) | 3.65  (2.01 to 5.73) | 2.81  (-0.87 to 6.5) | -5.32  (-10.4 to -0.23) | 0.01 |
| eGFR change in the first year (ml/min) | 2.31  (0.78 to 3.85) | -0.97  (-4.14 to 2.2) | -5.12  (-9.87 to -0.38) | 0.005 |
| eGFR change/y (ml/min/y) | -0.69  (-2.81 to 3.38) | 0.93  (-0.83 to 2.7) | -3.66  (-7.04 to -0.28) | 0.017 |
| eGFR decline >30% (%) | 11.8% | 20% | 32.3% | 0.003 |
| ESRD (%) | 9.4% | 20% | 25.8% | 0.003 |
| Combined end-point (%) | 14.8% | 24.3% | 32.3% | 0.01 |

***Abbreviations:*** *y, years; eGFR, estimated glomerular filtration rate; FU, follow-up; ESRD, end-stage renal disease.*
