# Supplementary material for: The impact of SARS-CoV-2 infection on renal function in patients with biopsy-proven kidney diseases
Source: PLoS One. 2023 Dec 22;18(12):e0296168. doi: 10.1371/journal.pone.0296168 (PMC10745175; doi:10.1371/journal.pone.0296168)
Supplement: S3 Table — (DOCX) [file pone.0296168.s005.docx]

**S3 Table. Renal function outcomes in relation to underlying etiology.**

| **Variable** | **Entire cohort** | **With COVID-19** | **Without COVID-19** | **p-value** | **Adjusted mean difference (95%CI)** | **p-value** |
| --- | --- | --- | --- | --- | --- | --- |
| **IgA Nephropathy** | | | | | | |
| Number of patients | 101 | 32 | 69 | - | - | - |
| eGFR at study onset (ml/min) | 49.1 ± 28.1 | 43.1 ± 26.2 | 51.8 ± 28.6 | 0.14 | - | - |
| eGFR at 12 months (ml/min) | 49.1 ± 30.3 | 43.9 ± 32.1 | 51.5 ± 29.4 | 0.19 | - | - |
| eGFR at last FU (ml/min) | 48.2 ± 30.2 | 40.9 ± 29.5 | 51.5 ± 30.1 | 0.09 | - | - |
| Total eGFR change (ml/min) | -0.85  (-2.66 to 0.96) | -2.15  (-5.48 to 1.17) | -0.24  (-2.44 to 1.95) | 0.23 | -1.72  (-5.66 to 2.22) | 0.38 |
| eGFR change in the first year (ml/min) | 0.11  (-1.84 to 2.08) | 0.87  (-3.21 to 4.96) | -0.23  (-2.24 to 2) | 0.86 | 1.33  (-2.96 to 5.63) | 0.54 |
| eGFR change/y (ml/min/y) | -0.5  (-1.43 to 0.43) | -1.06  (-2.95 to 0.81) | -0.23  (-1.32 to 0.84) | 0.22 | -0.69  (-2.73 to 1.33) | 0.49 |
| eGFR decline >30% (%) | 16.8% | 31.2% | 10.1% | 0.008 | - | - |
| ESRD (%) | 16.8% | 31.2% | 10.1% | 0.008 | - | - |
| Combined end-point (%) | 21.8% | 34.4% | 15.9% | 0.03 | - | - |
| **Membranous nephropathy** | | | | | | |
| Number of patients | 41 | 9 | 32 | - | - | - |
| eGFR at study onset (ml/min) | 67.8 ± 27.5 | 77.4 ± 30.1 | 65 ± 26.6 | 0.26 | - | - |
| eGFR at 12 months (ml/min) | 68.4 ± 29.8 | 71.6 ± 28.4 | 67.4 ± 30.5 | 0.7 | - | - |
| eGFR at last FU (ml/min) | 70.8 ± 29.3 | 73.3 ± 34.7 | 70.1 ± 28.2 | 0.76 | - | - |
| Total eGFR change (ml/min) | 3  (-1.7 to 7.74) | -4.11  (-14.4 to 6.2) | 5.06  (-0.35 to 10.4) | 0.1 | -8.3  (-19.6 to 2.91) | 0.14 |
| eGFR change in the first year (ml/min) | 0.6  (-3.9 to 5.1) | -5.77  (-20.7 to 9.1) | 2.45  (-1.91 to 6.81) | 0.25 | -7.9  (-18.8 to 3.01) | 0.15 |
| eGFR change/y (ml/min/y) | 1.44  (-0.65 to 3.54) | -1.81  (-6.45 to 2.83) | 2.38  (0.003 to 4.77) | 0.09 | -3.8  (-8.78 to 1.16) | 0.13 |
| eGFR decline >30% (%) | 4.9% | 11.1% | 3.1% | 0.39 | - | - |
| ESRD (%) | 0% | 0% | 0% | - | - | - |
| Combined end-point (%) | 4.9% | 11.1% | 3.1% | 0.39 | - | - |
| **Focal and segmental glomerulosclerosis** | | | | | | |
| Number of patients | 40 | 6 | 34 | - | - | - |
| eGFR at study onset (ml/min) | 54.2 ± 32.8 | 63.3 ± 45.9 | 52.4 ± 30.4 | 0.49 | - | - |
| eGFR at 12 months (ml/min) | 53.3 ± 29.2 | 48 ± 34.5 | 54.3 ± 28.6 | 0.68 | - | - |
| eGFR at last FU (ml/min) | 57 ± 33.8 | 48.6 ± 42.9 | 58.6 ± 32.3 | 0.78 | - | - |
| Total eGFR change (ml/min) | 2.81  (-6.25 to 11.8) | -14.6  (-34.3 to 4.96) | 6.1  (-3.89 to 16.2) | 0.05 | -16.3  (-38.9 to 6.2) | 0.15 |
| eGFR change in the first year (ml/min) | -0.91  (-6.97 to 5.14) | -15.3  (-29.6 to -0.98) | 1.87  (-4.64 to 8.38) | 0.006 | -14.6  (-28.8 to -0.4) | 0.04 |
| eGFR decline >30% (%) | 32.5% | 66.7% | 26.5% | 0.07 | - | - |
| ESRD (%) | 20% | 50% | 14.7% | 0.08 | - | - |
| Combined end-point (%) | 32.5% | 66.7% | 26.5% | 0.07 | - | - |
| **Lupus Nephritis** | | | | | | |
| Number of patients | 48 | 17 | 31 | - | - | - |
| eGFR at study onset (ml/min) | 72.7 ± 30.5 | 71.5 ± 31.4 | 73.3 ± 30.5 | 0.85 | - | - |
| eGFR at 12 months (ml/min) | 73.2 ± 30.9 | 68.8 ± 32.4 | 75.5 ± 30.3 | 0.49 | - | - |
| eGFR at last FU (ml/min) | 80.5 ± 33.7 | 85.3 ± 40.3 | 78 ± 29.9 | 0.51 | - | - |
| Total eGFR change (ml/min) | 7.86  (2.45 to 13.2) | 13.7  (3.96 to 23.5) | 4.62  (-1.92 to 11.1) | 0.11 | 9  (-2.1 to 20.1) | 0.11 |
| eGFR change in the first year (ml/min) | 0.48  (-3.98 to 4.96) | -2.64  (-10.3 to 4.9) | 2.2  (-3.55 to 7.97) | 0.29 | -5.07  (-14.2 to 4.1) | 0.27 |
| eGFR change/y (ml/min/y) | 3.29  (0.78 to 5.83) | 5.04  (0.1 to 9.9) | 2.33  (-0.68 to 5.35) | 0.33 | 2.66  (-2.67 to 8) | 0.32 |
| eGFR decline >30% (%) | 8.3% | 5.9% | 9.7% | 0.99 | - | - |
| ESRD (%) | 6.2% | 5.9% | 6.6% | 0.99 | - | - |
| Combined end-point (%) | 10.4% | 5.9% | 12.9% | 0.64 | - | - |

***Abbreviations:*** *y, years; eGFR, estimated glomerular filtration rate; FU, follow-up; ESRD, end-stage renal disease.*
