# Supplementary material for: The impact of SARS-CoV-2 infection on renal function in patients with biopsy-proven kidney diseases
Source: PLoS One. 2023 Dec 22;18(12):e0296168. doi: 10.1371/journal.pone.0296168 (PMC10745175; doi:10.1371/journal.pone.0296168)
Supplement: Attachment — Submitted filename: Response to Reviewers.docx [file pone.0296168.s006.docx]

Dear Plos One Editorial team,

On behalf of the co-authors, I want to thank you for the opportunity of incorporating editorial and reviewer comments made in relation to our manuscript entitled “The impact of SARS-CoV-2 infection on renal function in patients with biopsy-proven kidney diseases”. We hope to have addressed all the comments and suggestions and believe that it has made our report clearer and more meaningful for publication.

Sincerely,

Bogdan Obrisca

**Editor**

**The manuscript addresses a topic of potential interest; however, there are significant drawbacks in the study that need to be addressed to draw robust conclusions. To highlight some of these limitations, i) acknowledgement that the retrospective nature of the study introduces several confounding factors and raises questions about whether renal progression may be attributed to underlying renal diseases per se; ii) lack of indications on the proportion of patients in the positive group who discontinued therapy, and variations in immunosuppressive protocols could impact renal progression; iii) no information is provided regarding the systematic COVID-19 nucleic acid screening during follow-up time in the negative group; iv) absence of statistical evaluation of differences in 24-hour urinary protein between negative and positive patients during the follow-up time; iv) the number of endpoint events in COX regression should be at least 10 times greater than the number of independent variables, while here ESRD only occurred more than 30 times, so the independent variables of COX regression should be limited only to 3 to 4 terms.**

**Response: We appreciate the suggestions made by the editor and reviewers in relation to our manuscript. We hope that based on these suggestions, our revised manuscript is clearer and our results more meaningful. In addition, during the review process of this manuscript, two other studies regarding the relation of glomerular disorders with SARS-CoV-2 have been published and the reference list has been updated accordingly (Wang C et al, Association of COVID-19 Versus COVID-19 Vaccination With Kidney Function and Disease Activity in Primary Glomerular Disease: A Report of the Cure Glomerulonephropathy Study, *Am J Kid Dis;* Gauckler P et al, COVID-19 outcomes in patients with a history of immune mediated glomerular diseases, *Frontiers in Immunology*). However, as the CureGN cohort only includes patients with IgAN/MCD/FSGS/MN and the second study included only 59 patients with glomerular disorders and SARS-CoV-2 infection, we appreciate that our study included a cohort with a variety of patients with glomerular disorders that is representative of a real-world renal biopsy registry from a tertiary center and with a longer post-infection follow-up compared to previous studies (median 2.5 years compared to 0.8 years in the CureGN cohort).**

**Nonetheless, our study has several limitations as pointed out by the reviewers that need to be acknowledged. Accordingly, our study limitation section has been revised appropriately.**

**i) acknowledgement that the retrospective nature of the study introduces several confounding factors and raises questions about whether renal progression may be attributed to underlying renal diseases per se;**

**Response: We agree that the retrospective nature of the study might introduce potential confounding factors other than those adjusted for in the multivariate analysis, and we updated the limitations of the study section accordingly. However, in an attempt to overcome this aspect, we stratified the analysis according to immunosuppression use, type of underlying disorders (for the most frequent etiologies) and adjust in Cox regression for the confounding effect of IS and type of glomerular disorder on renal outcome. Moreover, given the widespread use of IS in this cohort (over 70%) and the known relation between treatment and glomerular disease activity, it is likely that the worsening of renal function may be related to COVID-19, irrespective of the underlying etiology.**

**ii) lack of indications on the proportion of patients in the positive group who discontinued therapy, and variations in immunosuppressive protocols could impact renal progression.**

**Response: Our center’s protocol was not to modify the immunosuppressive regimens during the COVID-19 pandemic. However, during a SARS-CoV-2 infection a temporarily reduction in the doses of immunosuppressive regimens (for up to 10-14 days following the resolution of the infection) was undertaken. We have mentioned this aspect in the methods section. Regarding the second part of the question, we agree that there is a significant heterogeneity of immunosuppression regimens used among different glomerular disorders. However, it is difficult to adequately evaluate the independent effect of each IS regimen. Given this limitation (that is encountered in any study enrolling patients from a biopsy registry), we attempted to stratify the analysis according to the use of immunosuppression showing that COVID-19 occurrence influenced renal function decline irrespective of immunosuppression use, albeit to a higher degree in those without immunosuppression exposure. We have also highlighted this aspect in the discussion.**

**iii) no information is provided regarding the systematic COVID-19 nucleic acid screening during follow-up time in the negative group.**

**Response: SARS-COV-2 infection screening was undertaken regularly in our center in this category of patients with a periodicity of 1-3 months. Our center’s protocol was to undertake a proactive screening for SARS-CoV-2 infection** in this category of immunosuppressed patients **via outpatient visits, hospital admissions, periodic telephone interviews and a thorough review of the electronic clinical health records during the study follow-up.**

**We have updated the methods section to describe this aspect.**

**iv) absence of statistical evaluation of differences in 24-hour urinary protein between negative and positive patients during the follow-up time.**

**Response: Indeed, proteinuria is one of the most significant risk factors for CKD progression irrespective of the underlying etiology. However, because of the retrospective nature of this study and the initial mobility restriction in the context of the pandemic, we had many missing proteinuria assessments. We acknowledge that this is a limitation of the study and updated this section accordingly. In addition, in a recent CureGN analysis (published during the review of this paper), the impact of SARS-CoV-2 of future GFR loss remained meaningful even after adjustment for baseline proteinuria. So, with this drawback, we still believe that the impact of SARS-CoV-2 on renal outcome would have remained significant even after proteinuria inclusion in the model. Moreover, given the suggestion to limit the number of variables in the Cox model, we would still have a limited number of variables to potentially adjust for. In addition, the relevance of proteinuria on renal outcomes may differ significantly between different glomerular disorders (e.g.: IgAN vs. MCD vs. MN).**

**v) the number of endpoint events in COX regression should be at least 10 times greater than the number of independent variables, while here ESRD only occurred more than 30 times, so the independent variables of COX regression should be limited only to 3 to 4 terms.**

**Response: We appreciate this suggestion. In this cohort, we had 53 cases of ESRD and 76 cases of the composite endpoint (ESRD and more than 30% decrease in eGFR). Accordingly, in order to select the best prediction models, we modified our multivariate Cox regression models and chose a stepwise backward elimination method. According to this model, we were left with 6 variables in the analysis regarding the composite endpoint and 4 variables in the analysis regarding only ESRD. We have modified throughout the manuscript this new analysis and updated the methods section to highlight the method used for multivariate Cox regression.**

**Reviewer 1**

The idea of the paper is interesting however I have some comments.

1. The study is retrospective which made many confounding factors to involve. The renal progression may be caused by underlying renal diseases per se. The different IS protocols may have impact on the renal progression, also any acute illness or other medications can have impact. How can the author explain this.

**Response:** We agree that the retrospective nature of the study might introduce potential confounding factors other than those adjusted for in the multivariate analysis, and we updated the limitations of the study section accordingly**.** We have tried to account for underlying disease in two ways. Firstly, by also analyzing the renal progression in the most frequent etiology categories. Second, we have adjusted in the multivariate analysis (Cox and linear regression) by the potential impact of the underlying etiology and use of immunosuppression. By this approach, we have identified that the impact of SARS-CoV-2 infection on renal function decline occurred independently of the underlying etiology and was most evident in patients with IgAN, FSGS and MN. In addition, we agree that given the variability of immunosuppression regimens used it is difficult to evaluate the independent effect of each regimen. However, given the known relation between treatment and the control of glomerular disease activity, it is likely that the accelerated renal function decline may be related to COVID-19, especially given the graded impact correlating with infection severity. Acknowledging these limitations, we have updated the limitation section of the study accordingly.

2.It appears that SARS-CoV-2 infection may have induced a form of AKI and even many sentences that specify the effect of SARS-COV-2 and kidney that implies to this study should have more proof and state in the manuscript.

**Response:** Thank you for the observation. Indeed, the relation between SARS-CoV-2 infection and AKI in our study was just a hypothesis given the observation that the maximal impact on GFR loss in this study was seen at 12 months (and in other studies at 6 months). However, we could not definitely prove this aspect due to the absence of AKI assessment episodes and lack of evaluation for potential AKI biomarkers. In agreement with this aspect, we have updated the discussion section accordingly.

**Reviewer 2**

**1. Was COVID-19 nucleic acid screening regularly performed during follow-up time (For example, every 2 weeks or at least 1 month) in the negative group. And how to determine whether the patients in the negative group were never infected with COVID-19 during the follow-up time?**

**Response:** **SARS-COV-2 infection screening was undertaken regularly in our center in this category of patients with a periodicity of 1-3 months. Our center’s protocol was to undertake a proactive screening for SARS-CoV-2 infection** in this category of immunosuppressed patients **via outpatient visits, hospital admissions, periodic telephone interviews and a thorough review of the electronic clinical health records during the study follow-up. Similar to previous studies, COVID-19 was defined as participants who reported “definitely” or “probably or suspected” COVID-19 or had a positive antigen or serological test for COVID-19. Patients that never had any suspicion for SARS-CoV-2 infection, had persistent negative testing and negative serologic work-up were considered SARS-CoV-2 negative. We have updated the methods section to describe this aspect.**

**2. Please describe the number and cause of death of patients in the two groups respectively.**

**Response: 19 patients died during the study follow-up: 3 patients due to SARS-CoV-2 infection and 16 patients due to reasons unrelated to COVID-19 (12 patients due to cardiovascular causes, 3 patients due to underlying malignancy and 1 patient due to a non-SARS-CoV-2 infection). We have updated this in the result section.**

**3. Please describe the proportion of patients in the positive group who discontinued immunosuppressive therapy.**

**Response: Our center’s protocol was not to modify the immunosuppressive regimens during the COVID-19 pandemic. However, during a SARS-CoV-2 infection a temporarily reduction in the doses of immunosuppressive regimens (for up to 10-14 days following the resolution of the infection) was undertaken. We have updated this aspect in the methods section.**

**4. Please describe the number of COVID-19 infections for each patient in the positive group.**

**Response:** **Nine patients were identified to have two SARS-CoV-2 infection during the study follow-up, which all the subsequent infection being mild in severity. We have added this information to the result section.**

**5. Were there significant differences in 24-hour urinary protein between the two groups during the follow-up time?**

**Response: Thank you for the observation. Indeed, proteinuria is one of the most significant risk factors for CKD progression irrespective of the underlying etiology. However, because of the retrospective nature of this study and the initial mobility restriction in the context of the pandemic we had many missing proteinuria assessments. We acknowledge that this is a limitation of the study and updated this section accordingly. In addition, in a recent CureGN analysis (published during the review of this paper), the impact of SARS-CoV-2 of future GFR loss remained meaningful even after adjustment for baseline proteinuria. So, with this drawback, we still believe that the impact of SARS-CoV-2 on renal outcome would have remained significant even after proteinuria inclusion in the model. Moreover, given the suggestion to limit the number of variables in the Cox model, we would still have a limited number of variables to potentially adjust for. In addition, the relevance of proteinuria on renal outcomes may differ significantly between different glomerular disorders (e.g.: IgAN vs. MCD vs. MN).**

**6. The number of endpoint events in COX regression should be at least 10 times greater than the number of independent variables. In this paper, ESRD only occurred more than 30 times, so the independent variables of COX regression should be limited only to 3 to 4 terms.**

**Response: We appreciate your suggestions. In this cohort, we had 53 cases of ESRD and 76 cases of the composite endpoint (ESRD and more than 30% decrease in eGFR). Accordingly, in order to select the best prediction models, we modified our multivariate Cox regression models and chose a stepwise backward elimination method. According to this model, we were left with 6 variables in the analysis regarding the composite endpoint and 4 variables in the analysis regarding only ESRD. We have modified throughout the manuscript this new analysis and updated the methods section to highlight the method used for multivariate Cox regression.**
