# Supplementary material for: The impact of SARS-CoV-2 infection on renal function in patients with biopsy-proven kidney diseases
Source: PLoS One. 2023 Dec 22;18(12):e0296168. doi: 10.1371/journal.pone.0296168 (PMC10745175; doi:10.1371/journal.pone.0296168)
Supplement: Attachment — Submitted filename: Response to Reviewers.docx [file pone.0296168.s007.docx]

Dear Plos One Editorial team,

On behalf of the co-authors, I want to thank you for the opportunity of incorporating editorial and reviewer comments made in relation to our revised manuscript entitled “The impact of SARS-CoV-2 infection on renal function in patients with biopsy-proven kidney diseases”. We hope to have addressed all the comments and suggestions and believe that it has made our report clearer and more meaningful for publication.

Sincerely,

Bogdan Obrisca

**Editor**

**The manuscript focuses on a topic of potential interest. While the study has been significantly improved, there are still some minor concerns that should be addressed. To mention some of them: i) explain better and report in the table the severity of disease; ii) indicate that the heterogeneity of the underlying pathology could make it difficult to evaluate the progression of renal disease; iii) the lack of urinalysis data, not only proteinuria, but also haematuria and/or leukocyturia that could limit the evaluation of the possible renal involvement in COVID-19 infection; iv) include the suggested references; v) define the acronyms in full at first mention and correct typos.**

**Response: We appreciate the suggestions made by the editor and reviewers in relation to our manuscript. We hope that based on these suggestions, our revised manuscript is clearer and our results more meaningful. Please find below our answers to the issues raised.**

**i) The SARS-CoV-2 infection severity was assessed as defined by the COVID-19 Treatment Guidelines Panel of the National Institutes of Health, and detailed accordingly in the methods section. In our study we have distinctly evaluated the impact of infection severity on renal outcomes (mild-moderate versus severe COVID-19) and detailed it in Supplemental Table II. In addition, the Cox regression analysis was done to evaluate the impact of mild-moderate infection versus no infection and severe infection versus no infection. To be clearer in the conclusion of our study we have modified it as:**

**“In conclusion, we have identified a significant impact of SARS-CoV-2 infection on long-term renal function in patients with biopsy-proven kidney diseases that leads to a greater decline of eGFR and a worse renal survival, with a graded impact correlating with infection severity, those with severe forms of COVID-19 having the worst renal outcome.”**

**ii) We have updated the limitations section indicating that the heterogeneity of the underlying pathology could bring limitations to evaluation of the renal disease progression.**

**iii) Thank you for the observation. We have included this aspect in the limitations of the study section. However, as previously mentioned by the reviewers, we could not have been able to include the urinalysis in the multivariate Cox regression due to the suggestion to limit our variables included in the model. Nonetheless, the stratification of the analysis according to the underlying disorders and use of immunosuppression provided the same results, and we feel that this may be a surrogate marker of the underlying activity of the disease.**

**iv) We have included the suggested references.**

**v) We have revised the entire manuscript for typing errors and acronyms.**

**We hope that we have addressed all the issues raised.**

**Reviewer 3**

**1) Clinically this study may be interesting even if it presents several limitations already inserted by the authors, as requested by the reviewers, but which may make the correct evaluation of the end points difficult.**

**In addition to the retrospective aspect, widely underlined by the reviewers and authors in the manuscript, the heterogeneity of the underlying pathology could also make it difficult to evaluate the progression of renal disease.**

**Response: We have updated the limitations section indicating that the heterogeneity of the underlying pathology could bring limitations to evaluation of the renal disease progression. However, apart from the CureGN cohort that included only patients with MCD/FSGS/MN/IgAN, the rest of the population-based studies have evaluated patients with CKD based solely on eGFR and/or albuminuria, while a heterogeneity of an underlying pathology might be also present in these cases (but not taken into account due to the lack of etiological assessment). Despite being clearly a limitation of the study, we believe that our attempt provides a better characterization of the study population compared to previous studies.**

**2) Furthermore, the lack of urinalysis data, not only proteinuria, but also hematuria and/or leukocyturia that could help to evaluate the possible renal involvement in COVID 19 infection.**

**Response: Thank you for the observation. We have included this aspect in the limitations of the study section. However, as previously mentioned by the reviewers, we could not have been able to include the urinalysis in the multivariate Cox regression due to the suggestion to limit our variables included in the model. Nonetheless, the stratification of the analysis according to the underlying disorders and use of immunosuppression provided the same results, and we feel that this may be a surrogate marker of the underlying activity of the disease.**

**3) …….This study, the first to our knowledge to address the long-term impact of SARS-CoV-2 infection on renal function:……**

**-Mohammad Atiquzzaman, Jordyn R Thompson, Selena Shao, Ognjenka Djurdjev, Micheli Bevilacqua, Michelle M Y Wong, Adeera Levin, Peter C Birks. Long-term effect of COVID-19 infection on kidney function among COVID-19 patients followed in post-COVID recovery clinic in British Columbia, Canada. Nephrol Dial Transplant . 2023 Jun 22:gfad121. doi: 10.1093/ndt/gfad121. Online ahead of print.**

**Response: Thank you for the suggested reference that we included in the discussion section. However, this cohort had a mean eGFR of 88 ml/min, while less than 25% of the study cohort had an eGFR below 60 ml/min, much lower than the mean 52 ml/min in our study, suggesting that we included a population with a higher risk for CKD progression. Nonetheless, similar to our study, this reference highlights the risk of eGFR loss within the first year following COVID-19 and this strengthens our similar observations. In addition, apart from the CureGN cohort that included a limited number of underlying CKD pathology, our study remains the first one to include a well characterized patient population with regards to underlying pathology, that rather reflects a population typical for a tertiary clinic.**

**4) The acronyms should be written in full first, there are some typos.**

**Response: We have revised the entire manuscript for typing errors and acronyms.**

**5) In conclusion, we have identified a significant impact of SARS-CoV-2 infection on long term renal function in patients with biopsy-proven kidney diseases that leads to a greater decline of eGFR and a worse renal survival, with a graded impact correlating with infection severity…………**

**The authors should explain better and report in the table the severity of disease.**

**Response: The SARS-CoV-2 infection severity was assessed as defined by the COVID-19 Treatment Guidelines Panel of the National Institutes of Health, and detailed accordingly in the methods section. In our study we have distinctly evaluated the impact of infection severity on renal outcomes (mild-moderate versus severe COVID-19) and detailed it in Supplemental Table II. In addition, the Cox regression analysis was done to evaluate the impact of mild-moderate infection versus no infection and severe infection versus no infection. To be clearer in the conclusion of our study we have modified it as:**

**“*In conclusion, we have identified a significant impact of SARS-CoV-2 infection on long-term renal function in patients with biopsy-proven kidney diseases that leads to a greater decline of eGFR and a worse renal survival, with a graded impact correlating with infection severity, those with severe forms of COVID-19 having the worst renal outcome*”**

**We hope that this addressed your issue.**

**6) Should be interesting to insert the data about serum electrolytes and acid-base balance over time to evaluate a possible association between COVID infection and CKD progression.**

**Response: Although we do agree that evolution of electrolytes and acid-base balance would have been very interesting to assess, the retrospective nature of the study and the fact that some patients were followed remotely during the lock-down period makes very difficult to collect this huge amount of data. In addition, many patients were already excluded from the study due to a lack of an adequate assessment of renal function due to various reasons. Including electrolyte/acid-base assessment would have further limited the number of patients that would have been eligible for study inclusion. However, we agree that this aspect would be very interesting and ideal to be assessed in a prospective cohort.**

**7) Should be interesting to insert the inflammatory indexes which are generally associated with Covid 19 infection but can also be increased in CKD, and can persist even in long COVID.**

**-The PHOSP-COVID Collaborative Group. Clinical characteristics with inflammation profiling of long COVID and association with 1-year recovery following hospitalisation in the UK: a prospective observational study Lancet Respir Med. 2022 Aug; 10(8): 761–775.**

**Response:** Thank you for the suggestion and we included this reference in the discussion section. However, similar to the previous response, the retrospective nature of the study and the fact that some patients were followed remotely during the lock-down period makes it very difficult to collect this nature of data.
